# Estimation of stature from finger and phalange lengths in a Korean adolescent

**DOI:** 10.1186/s40101-019-0206-1

**Published:** 2019-10-22

**Authors:** Ilsun Rhiu, Wonjoon Kim

**Affiliations:** 1 Division of Big Data and Management Engineering, Hoseo University, Asan, 31499 South Korea; 2 Department of Industrial and Management Engineering, Sungkyul University, Anyang, 14097 South Korea

## Background

The major goal of a forensic investigation is to verify identity through the victim’s remains [1], which consists of individual parts of the body that are ashes, damaged, or dismembered. As such, partially damaged bodies and human remains are often encountered in fields, where intentional cutting, dismantling, explosion, or other mass disasters took place. The main purpose of investigating the damaged remains is to develop a biological profile that identifies individuals by estimating the age, gender, and stature of the remains [2, 3]. This profile helps to increase the likelihood of identifying the information of the victim’s events or disasters.

Estimating statures through human remains is based on the principle that there is a linear relationship between statures and various parts of the human body and bones [4]. Previous studies that estimate statures through different parts of the body have been shown to be able to structure an estimation model with reasonable accuracy. Ahmed [5] designed a model to estimate stature and sex using upper arm length, ulnar length, and wrist breadth associated with the upper limb. Ozden et al. [6] estimated stature using the measurements of foot and shoe. Pelin et al. [7] performed a study to estimate stature from head and face dimensions. Anthropometric studies have been actively conducted to estimate statures using the upper limbs, lower extremities, and hands. Previous studies have shown that estimating statures can be more accurately estimated by using longer segments such as the upper and lower limbs of various parts of the body [5, 8].

However, much research has not been done to estimate statures through the fingers and phalanges. In previous studies that estimated human characteristics such as gender and stature through fingers and phalanges, Rastogi et al. [9] predicted statures through the length of the middle finger for the Indians. Kanchan and Rastogi [10] used an index and a ring finger to design a model to determine the gender of an Indian. Habib and Kamal [11] estimated stature based on the length of the hand and the length of the phalanges of each finger. Jasuja and Singh [12] estimated stature by using the measurement variables of finger and phalange of North Indian adults. Agrawal et al. [13] designed a model that estimates stature by using the length of the hand and phalanges of North Indians.

Most of the studies predicting statures by measuring fingers and phalanges were performed on adults. Krishan et al. [14] estimated stature in the northern Indian adolescent through the length of their index and ring fingers, and Ibegbu et al. [15] designed a regression model that estimated stature through the hand length in children of Nigeria. However, in many previous studies, it was confirmed that the results of stature estimation by anthropometry differed by ethnicity. Therefore, it is necessary to study the estimation of stature through the finger and phalange lengths of Korean adolescents.

From the point of view of forensic research, in case of an unresolved murder or a devastation disaster, such as a tsunami, research is needed to help estimate the stature of a person through various body parts to establish the biological profile of an unidentified individual. Especially, in case of such an affair or accident, it is difficult for the body part to be maintained in an intact state. In addition, in the case of remains caused by murder, it is likely that it has been intentionally damaged to conceal the identity of the victim. Therefore, the study of designing criteria for establishing biological profiles is very important. Based on these studies, it is possible to identify people who cannot be identified.

Most previous studies estimating stature through hand measurement related to the entire shape of the hand such as hand length and hand breadth. However, it can be difficult to get the victim’s hands in intact condition at various crime scenes. In addition, research on estimating stature through various parts of the body has been conducted on various ethnicities, but only few studies were conducted on Koreans. In addition, most studies on Koreans, as well as other ethnic groups, have conducted adults [16]. In recent years, crimes against adolescents have been increasing, and as the methods have become diverse and cruel, research on the estimation of stature is needed.

The purpose of this study was to investigate the relationship between the stature and the length of fingers and phalanges in the Korean adolescent population. This study derives a linear regression model for stature estimation from 5 fingers and 14 phalanges. In addition, regression models with the highest prediction accuracy according to gender were derived from various regression models.

## Methods

### Subjects

For this study, the subjects were recruited to Koreans born in South Korea. Since there is a time-dependent variation in human stature, all measurements were made at once in the morning. Therefore, in order to unify these measurement considerations, adolescents living in Seoul, the capital of South Korea, were selected as subjects. Five subjects (males 3, females 2) with vertebral-related deformities or congenital camptodactyly were excluded from this study. Finally, a total of 172 subjects (males 89, females 83) participated in the measurements, and the subjects were between 14 and 18 years of age. Their average age was 15.78 years for males (SD = 1.41) and 15.88 years for females (SD = 1.46). All subjects were also right-handed for consistency of measurement. This study was approved by the research ethics committee of Seoul National University (SNUIRB no. E1505/001–002) and was, therefore, conducted according to the guidelines laid down in the Declaration of Helsinki.

### Measurements

**Fig. 1:**
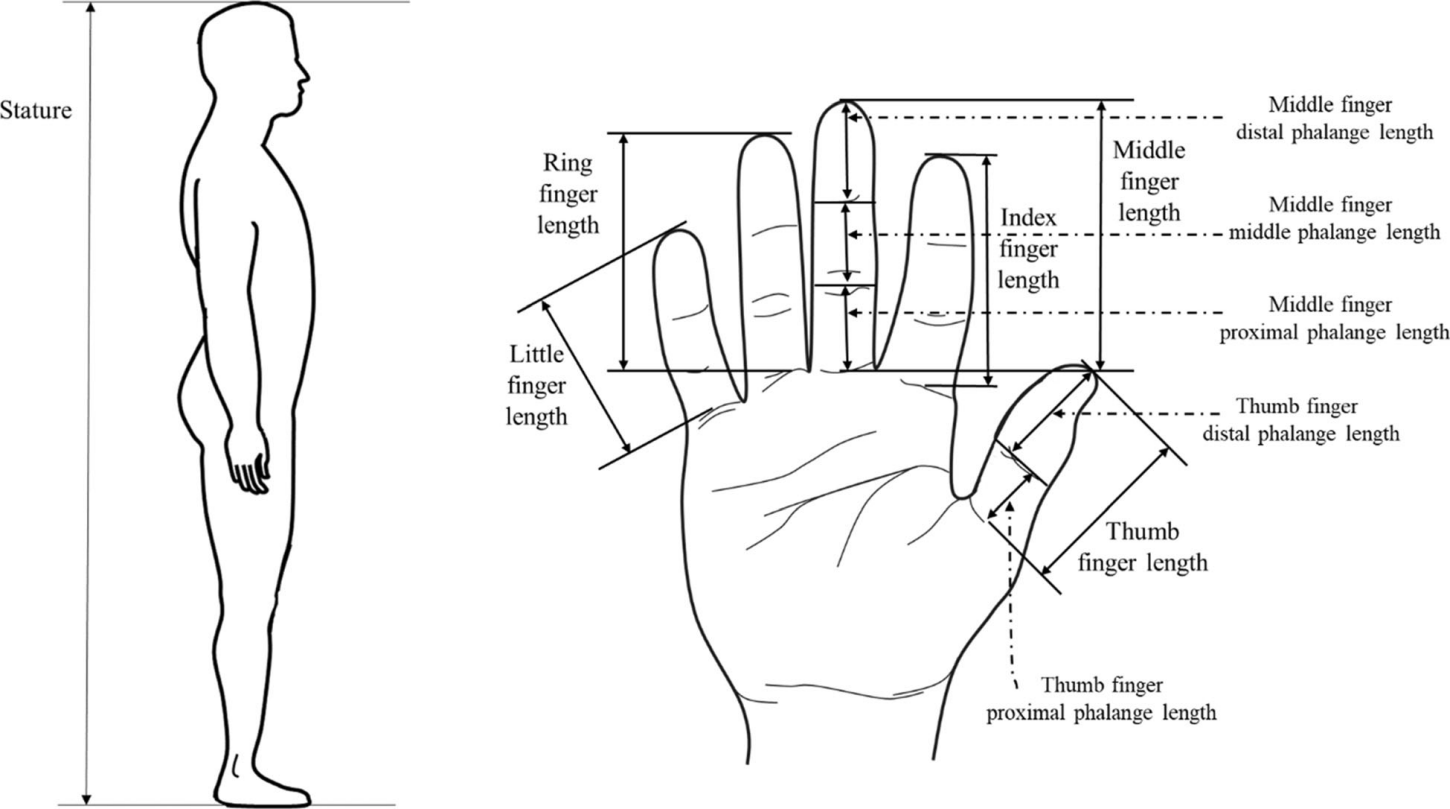
Schematic illustration of measurements in this study

Anthropometric measurements of the statures and fingers were performed following Vallois [17]. For the consistency of measurements, finger measurements were performed on the right hand only (see Fig. 1). All measurements were made using a Martin anthropometer and a caliper in indicated by centimeters and millimeters (TTM, Japan). Information on the measurement variables for the lengths of finger and phalange is shown in Table 1 below. For stature measurement, an anthropometer was used in this study. Stature is measured as the vertical distance between the vertex point of the head and the floor surface. The subject is placed in parallel with the anthropometer and stands straight in a frontal gaze position. An investigator recorded the stature of the subjects indicated on the anthropometer. The length of the fingers was measured from the midpoint of the proximal crease of each finger to the tip of each finger using a caliper. Three types of phalange: proximal, middle, and distal were selected as the measurement variables in this study, and the method of measuring each phalange are described in Table 1.

**Table 1:** Definition of the finger and phalange measurement [18]

Category	Measurement variable	Abbreviation	Description
Finger	Thumb, index, middle, ring, little finger length	1DL, 2DL, 3DL, 4DL, 5DL	The distance from proximal flexion crease of the finger to the tip of the respected finger
Phalange	Thumb, index, middle, ring, little finger proximal phalange length	1DT2L, 2DI3L, 3DM3L, 4DR3L, 5DL3L	The distance from the proximal interphalangeal joint crease to metacarpophalangeal joint crease of each finger
	Index, middle, ring, little finger middle phalange length	2DI2L, 3DM2L, 4DR2L, 5DL2L	The distance from the distal interphalangeal joint crease to the proximal interphalangeal joint crease
	Thumb, index, middle, ring, little finger distal phalange length	1DT1L, 2DI1L, 3DM1L, 4DR1L, 5DL1L	The distance from the most forwarding projecting point on the tip of each finger to distal interphalangeal joint crease of each finger

During the acquisition of the measurement data, the accuracy of the measurements was checked periodically. All measurements were performed by one trained anthropometric person. Before performing this measurement, the investigator performed preliminary measurements on 12 subjects (males = 6, females = 6). Stature, finger, and phalange lengths were measured two times for each subject. Intra-observer error about each variable was measured by the technical error of measurement, relative technical error of measurement (rTEM), and coefficient of reliability (*R*). The results are shown in Table 2 below. According to Ulijaszek and Kerr [19], if the value of rTEM is lower than 5%, the intra-observer error is regarded as an acceptable standard range for all measurements. In terms of the coefficient of reliability, Goto [20] argued that if the coefficient of reliability was above 0.95, the quality of measurement was good controlled. Therefore, it is confirmed that the preliminary measurement result and the technical error do not affect the whole measurement, and the measured result can be considered to have reliability without significant technical and measurement error.

**Table 2:** Results of TEM, rTEM (%), *R* calculation for each measurement variable (cm)

Category	Mean ± SD		TEM	rTEM (%)	*R*
	Time 1	Time 2			
Stature	167.967 ± 8.619	166.900 ± 8.438	8.052	0.481	0.991
1DL	5.835 ± 0.585	5.821 ± 0.589	0.483	0.829	0.993
2DL	6.887 ± 0.443	6.888 ± 0.476	0.656	0.953	0.980
3DL	7.722 ± 0.467	7.717 ± 0.479	0.666	0.863	0.979
4DL	7.135 ± 0.485	7.142 ± 0.486	0.617	0.865	0.982
5DL	5.662 ± 0.366	5.684 ± 0.372	0.475	0.837	0.984
1DT2L	2.870 ± 0.490	2.857 ± 0.534	0.510	1.781	0.993
2DI3L	2.274 ± 0.199	2.233 ± 0.215	0.430	1.910	0.977
3DM3L	2.596 ± 0.237	2.592 ± 0.231	0.360	1.389	0.975
4DR3L	2.192 ± 0.344	2.214 ± 0.337	0.506	2.298	0.978
5DL3L	1.754 ± 0.197	1.786 ± 0.205	0.427	2.412	0.966
1DT1L	3.089 ± 0.226	3.101 ± 0.214	0.313	1.011	0.981
2DI1L	2.216 ± 0.194	2.163 ± 0.168	0.536	2.449	0.962
3DM1L	2.608 ± 0.245	2.597 ± 0.233	0.361	1.386	0.977
4DR1L	2.352 ± 0.185	2.385 ± 0.179	0.360	1.522	0.975
5DL1L	1.598 ± 0.144	1.566 ± 0.122	0.327	2.070	0.978
2DI2L	2.424 ± 0.213	2.430 ± 0.220	0.410	1.690	0.962
3DM2L	2.494 ± 0.234	2.512 ± 0.243	0.363	1.448	0.979
4DR2L	2.523 ± 0.253	2.558 ± 0.261	0.363	1.429	0.989
5DL2L	2.285 ± 0.176	2.292 ± 0.182	0.279	1.221	0.975

### Statistical analysis

All statistical analyses were performed in R 3.4.4 (R Development Core Team, 2018). Gender differences in each length of finger and phalange, and stature were determined by *t* test. Correlation between stature and each length of finger and phalange was determined through Pearson correlation coefficient. Two types of regression analysis: simple and multiple were used to estimate stature. The regression equation was used to estimate stature from the length of each finger and phalange of males and females. The stature was used as a dependent variable and the length of fingers and phalanges as an independent variable. In addition, in this study, 10-fold cross-validation was used to derive a more robust regression equation. Therefore, in this paper, the average values of coefficient of determination (*R*^2^), and standard error of estimate (S.E.E) derived from 10 datasets were reported. Variables with a *p* value less than 0.05 were considered statistically significant.

## Results

### Descriptive statistic

The results of the descriptive statistics on the stature, finger, and phalanges between males and females are shown in Table 3 below. First, in the case of the stature, the range of males was between 152.413 cm and 188.368 cm, and that of females was between 150.039 cm and 171.329 cm. As can be seen in Table 3, in all measured variables, males showed higher values than females. The most significant difference between females and males was the length of the middle finger (*t* = 10.408). In the phalanges, the distal phalange of index and the distal phalange of thumb were the biggest difference (*t* = 8.513, 8.304).

**Table 3:** Descriptive statistics for stature, finger, and phalange length measurements (cm) in both genders

Measurement variable	Males (*n* = 89)			Females (*n* = 83)			*t* test	
	Mean	SE	SD	Mean	SE	SD	*t*	*p*
Stature	171.783	6.783	7.19	160.219	5.716	6.03	12.339	.000
1DL	6.011	0.392	0.42	5.490	0.380	0.40	9.041	.000
2DL	6.972	0.873	0.93	6.566	0.381	0.40	4.042	.000
3DL	7.938	0.456	0.48	7.287	0.379	0.39	10.408	.000
4DL	7.431	0.409	0.43	6.825	0.383	0.40	10.233	.000
5DL	5.849	0.372	0.39	5.382	0.396	0.42	8.141	.000
1DT2L	2.894	0.333	0.35	2.644	0.276	0.29	5.466	.000
2DI3L	2.274	0.236	0.25	2.163	0.199	0.21	3.401	.000
3DM3L	2.649	0.237	0.25	2.457	0.188	0.20	6.020	.000
4DR3L	2.326	0.224	0.24	2.176	0.180	0.19	4.943	.000
5DL3L	1.799	0.182	0.19	1.699	0.180	0.19	3.664	.000
1DT1L	3.235	0.246	0.26	2.959	0.196	0.21	8.304	.000
2DI1L	2.419	0.836	0.89	2.296	0.208	0.22	1.361	.000
3DM1L	2.601	0.235	0.25	2.368	0.217	0.23	6.897	.000
4DR1L	2.643	0.192	0.20	2.385	0.214	0.23	8.513	.000
5DL1L	2.395	0.202	0.21	2.160	0.213	0.22	7.574	.000
2DI2L	2.279	0.205	0.22	2.107	0.196	0.21	5.748	.000
3DM2L	2.688	0.222	0.24	2.462	0.193	0.20	7.270	.000
4DR2L	2.462	0.194	0.21	2.264	0.201	0.21	6.679	.000
5DL2L	1.655	0.183	0.19	1.523	0.199	0.21	4.646	.000

### Correlation analysis

There was a statistically significant correlation between the length of finger and phalange and stature. The Pearson correlation coefficients between the stature and the finger and phalange of males and females are shown in Table 4 below. In the case of males, the correlation coefficient was the highest in the index finger (*r* = .769). The correlation coefficients of the three parts of the phalange were highest in 3DM3L, 1DT1L, and 4DR2L, respectively (*r* = .544, .527, .555). In case of females, the correlation coefficient was the highest in the middle finger (*r* = .603). The correlation coefficients of the three parts of the phalange were highest in 1DT2L, 3DM1L, and 3DM2L, respectively (*r* = .452, .492, .387).

**Table 4:** Pearson correlation between stature and finger/phalange measurements in both genders

Measurement variable		Males	Females
Finger	1DL	.659**	.584**
	2DL	.769**	.536**
	3DL	.701**	.603**
	4DL	.732**	.488**
	5DL	.485**	.293**
Proximal phalange	1DT2L	.440**	.452**
	2DI3L	.500**	.333**
	3DM3L	.544**	.214
	4DR3L	.503**	.149
	5DL3L	.258*	.018
Distal phalange	1DT1L	.527**	.477**
	2DI1L	.446**	.365**
	3DM1L	.278**	.492**
	4DR1L	.386**	.444**
	5DL1L	.276**	.384**
Middle phalange	2DI2L	.520**	.291**
	3DM2L	.545**	.387**
	4DR2L	.555**	.296**
	5DL2L	.415**	.141

**Correlation is significant at the 0.01 level; *Correlation is significant at the 0.05 level

### Simple linear regression analysis

In this study, the estimation of stature in Korean adolescent is performed using linear regression analysis. First, the results of simple linear regression analysis are shown in Tables 5 and 6 below. For adolescent males, index finger (2DL) (*R*^2^ = .591, S.E.E = 4.308 cm), and ring finger (4DL) (*R*^2^ = .536, S.E.E = 4.588 cm) was confirmed to be the highest determining variables in the regression equation. Among the phalanges, length of ring finger middle phalange (4DR2L) showed the highest value of the coefficient of determination (*R*^2^ = .309, S.E.E = 5.598 cm). For adolescent females, middle finger (3DL) (*R*^2^ = .341, S.E.E = 4.314 cm), and thumb finger (1DL) (*R*^2^ = .364, S.E.E = 4.239 cm) was showed to be highest determining variables in the estimation of stature. Among the phalanges, length of middle finger proximal phalange (3DM1L) showed the highest value of the coefficient of determination (*R*^2^ = .242, S.E.E = 4.629 cm).

**Table 5:** Results of simple linear regression for estimation of stature (cm) in males

Males	Equation	*R* ^2^	S.E.E (cm)
1DL	*S* = 102.377 + 11.540(1DL)	.434	5.064
2DL	*S* = 90.088 + 11.574(2DL)	.591	4.308
3DL	*S* = 88.657 + 10.463(3DL)	.491	4.803
4DL	*S* = 80.088 + 12.337(4DL)	.536	4.588
5DL	*S* = 120.045 + 8.834(5DL)	.235	5.887
1DT2L	*S* = 146.194 + 8.803(1DT2L)	.193	6.047
2DI3L	*S* = 139.581 + 14.115(2DI3L)	.250	5.832
3DM3L	*S* = 131.354 + 15.206(3DM3L)	.296	5.649
4DR3L	*S* = 136.661 + 15.067(4DR3L)	.253	5.819
5DL3L	*S* = 154.354 + 9.638(5DL3L)	.067	6.505
1DT1L	*S* = 125.646 + 14.224(1DT1L)	.278	5.720
2DI1L	*S* = 143.101 + 11.412(2DI1L)	.199	6.026
3DM1L	*S* = 150.720 + 8.051(3DM1L)	.077	6.468
4DR1L	*S* = 135.379 + 13.733(4DR1L)	.149	6.209
5DL1L	*S* = 149.693 + 9.168(5DL1L)	.076	6.471
2DI2L	*S* = 132.990 + 16.985(2DI2L)	.270	5.751
3DM2L	*S* = 127.033 + 16.616(3DM2L)	.297	5.644
4DR2L	*S* = 123.459 + 19.584(4DR2L)	.309	5.598
5DL2L	*S* = 146.758 + 15.039(5DL2L)	.172	6.127

**Table 6:** Results of simple linear regression for estimation of stature (cm) in females

Females	Equation	*R* ^2^	S.E.E (cm)
1DL	*S* = 109.399 + 9.282(1DL)	.341	4.314
2DL	*S* = 109.385 + 7.757(2DL)	.288	4.487
3DL	*S* = 98.476 + 8.481(3DL)	.364	4.239
4DL	*S* = 112.621 + 6.989 (4DL)	.238	4.639
5DL	*S* = 138.889 + 3.997(5DL)	.089	5.083
1DT2L	*S* = 137.837 + 8.560(1DT2L)	.204	4.743
2DI3L	*S* = 140.863 + 9.036(2DI3L)	.111	5.012
3DM3L	*S* = 145.074 + 6.253(3DM3L)	.046	5.193
4DR3L	S = 150.894 + 4.392(4DR3L)	.022	5.257
5DL3L	*S* = 159.592 + .518(5DL3L)	.000	5.315
1DT1L	*S* = 120.675 + 13.393(1DT1L)	.228	4.672
2DI1L	*S* = 138.886 + 9.365(2DI1L)	.133	4.950
3DM1L	*S* = 130.364 + 12.642(3DM1L)	.242	4.629
4DR1L	*S* = 134.033 + 11.036(4DR1L)	.197	4.763
5DL1L	*S* = 139.903 + 9.474(5DL1L)	.147	4.908
2DI2L	*S* = 143.580 + 8.0004(2DI2L)	.085	5.086
3DM2L	*S* = 133.689 + 10.862(3DM2L)	.150	4.901
4DR2L	*S* = 142.671 + 7.846(4DR2L)	.088	5.077
5DL2L	*S* = 154.795 + 3.723(5DL2L)	.020	5.263

### Multiple linear regression analysis

In order to derive a regression equation with a higher coefficient of determination, multiple regression analysis was performed by combining the variables of the length of finger and phalange (Table 7). For each regression analysis, the stepwise method was used as mentioned above. First, the regression equation derived from the length of the fingers is as follows. In the case of adolescent males, the regression model consisted of three fingers (thumb, index, and ring) (*R*^2^ = .646, S.E.E = 4.055 cm).

**Table 7:** Results of multiple linear regression for estimation of stature (cm) from measurements

Measurement variable	Gender	Equation	*R* ^2^	S.E.E (cm)
Finger	Males	*S* = 72.595 + 5.896(2DL) + 5.035(4DL) + 3.355(1DL)	.646	4.055
	Females	*S* = 93.653 + 5.895(1DL) + 4.704(3DL)	.421	4.072
Phalange	Males	*S* = 85.042 + 11.311(2DI3L) + 10.956(3DM1L) + 12.876(2DI2L)	.615	4.226
	Females	*S* = 97.477 + 7.236(3DM3L) + 5.325(1DT1L) + 5.730(3DM2L) + 5.906(1DT2L)	.445	4.037
Finger and phalange	Males	S = 72.739 + 5.798(1DL) + 9.639(2DI1L) + 8.724(2DI2L) + 8.105(3DM3L)	.659	4.004
	Females	S = 89.439 + 6.642(1DL) + 9.283(4DR1L) − 10.480(5DL1L) + 4.872(3DM2L) + 8.432(3DM3L)	.529	3.741

Next, the results of multiple regression analysis for only the phalange variables were used. In the case of adolescent males, three types of phalanges: 2DI3L, 3DM1L, and 2DI2L were included in the regression equation (*R*^2^ = .615, S.E.E = 4.226 cm). In the case of adolescent females, the coefficient of determination of the regression equation was .445, and it was confirmed that four variables were included (3DM3L, 1DT1L, 3DM2L, and 1DT2L).

Finally, the results of regression equations including both the length of fingers and phalanges are shown. For adolescent males, the combination of the thumb finger length (1DL), index finger distal phalange length (2DI1L), index finger middle phalange length (2DI2L), and middle finger proximal phalange (3DM3L) derived an *R*^2^ value of 0.659 with an average estimation error of 4.004 cm, which was the highest coefficient of determination of the regression equation found in this study. For adolescent females, the combination of thumb finger length (1DL), ring finger distal phalange (4DR1L), little finger distal phalange length (5DL1L), middle finger middle phalange (3DM2L), and middle finger proximal phalange length (3DM3L) yielded an *R*^2^ value of 0.529 with an average estimation error of 3.741 cm, which was the smallest estimation error of the regression equation found in this study.

## Discussion

Through this study, it was confirmed that the stature of males in Korean adolescents was statistically significantly higher than that of females. In addition, all of the lengths and phalanges of each finger was also found to be statistically significantly larger in males. A variety of previous studies of different ethnicities have shown similar results to this study. It has been reported that the hand-related measurement variables of males in Mauritius [21], North and South India, Turkey [22], Egypt [11], and Slovakia [23] are larger than those of females. According to Kanchan et al. [24], it has been confirmed that in the case of adolescents in South India, the length of ring finger in males is significantly longer than the females. Krishan et al. [14] confirmed that the length of the index and ring finger of adolescent males in North India are significantly longer than females. Most sexual size dimorphism in human statures occurs due to gender differences in growth duration and rate during adolescent growth spurts [25]. Through this study, it was confirmed that males have grown physically larger than females in Korean adolescent population.

In this study, it was confirmed that the length of the finger and phalange had a significant correlation with stature. In a study of adolescents, Krishan reported a correlation coefficient of .748 between the index finger and stature of males and a correlation coefficient of .531 for females [14]. In previous studies that revealed the correlation between the body parts and the stature in the adolescent, Monyeki and Sekhotha [26] found that the correlation coefficients between arm span and stature of South African students aged 15–18 years were 0.76 for males and 0.76 for females. Krishan and Kumar [27] examine the correlation between cephalo-facial measurement and stature in Koli males of North India. They found that among the various cephalo-facial measurements, horizontal circumference of head had the highest correlation coefficient with height (*r* = 0.773). Shah et al. [28] showed that there is a positive correlation between head length and stature in adolescents of West India. In case of western Indian, the correlation coefficient between stature and head length was 0.26 for males and 0.69 for females.

Similar results have been reported in studies of adults. Previous studies have been conducted to estimate statures using fingers and phalanges in adults of various ethnicities [11–13]. Rastogi et al. confirmed that there is a positive correlation between the middle finger length and the stature [9], and Jee and Yun [18] found that there is a positive correlation between each finger and the stature of Korean adults. The purpose of this comparison is to ascertain whether the correlation between stature in the adolescent population and the length of the finger and phalange is like that of the adult population in Korea. Comparing the values of the correlations measured in this study with that of Jee and Yun [18], in Korean adults, the middle finger of males and the ring finger for females were the most correlated with stature. The results showed that the degree of correlation coefficient between finger length and stature was different for adults and adolescents in Koreans. In their study, the Pearson correlation coefficient ranged from .390 to .507 for males and .375 to .465 for females. Therefore, the correlation between the length of fingers and phalanges and the stature is more significant in the adolescent populations of Koreans than in adults. This contrasts with the results of other ethnicities, especially Indians. Through this study, it was confirmed that the correlations between finger length and height of various ethnicities in adolescence were different.

Comparing the regression models derived for stature estimation in this study, it was observed that the accuracy of predicted stature estimation in both regression models was higher for males than for females. The results of this study are like those of studies conducted on various ethnicities (Table 8). In case of Korean male adolescents, the coefficient of determination of the regression equation using the hand and phalange was higher than that of adults in Egypt, India, Iran, and Korea and adolescents in India. In the case of Korean female adolescents, the coefficient of determination of the regression equation using the hand and phalange was higher than that of adults in Egypt and Korea but lower than that of India and Iran.

**Table 8:** Comparison of the regression equation, *R*^2^, and S.E.E from previous studies

Author	Ethnicity	Gender	Equation	*R* ^2^	S.E.E (cm)
Krishan et al.	Indian (adolescent)	Males	*S* = 71.403 + 12.948(2DL)	.560	5.41
		Females	*S* = 122.437 + 4.559(4DL)	.367	4.79
Akhlaghi et al.	Iranian (adult)	Males	*S* = 108.718 + 0.834(3DL)	.454	–
		Females	*S* = 95.058 + 0.901(3DL)	.415	–
Jasuja and Singh	Indian (adult)	Males	*S* = 171.902 + 1.645(3DM1L)	.464	5.40
		Females	*S* = 152.07 + 3.446(2DI2L)	.387	5.22
Jee and Yun	Korean (adult)	Males	*S* = 111.159 + 7.44(3DL)	.301	5.34
		Females	*S* = 107.152 + 6.98(4DL)	.211	5.71

In forensic research, various studies have been conducted to derive a formula for estimating the stature of a specific ethnicity using a variety of anthropometric data, such as upper and lower limb, and facial measurement. Milašinović et al. [29] found that the coefficient of determination between arm span and stature of adolescents in Montenegro was 0.673 for males. According to Ibegbu et al. [15], the coefficient of determination between hand length and stature of Nigerian students was 0.498 for males and 0.494 for females. Krishan et al. [30] designed a model that estimates the stature of adolescent males in North India through the measurement variables associated with the foot length. The coefficients of determination of the models were from 0.613 to 0.666.

Looking at previous studies, there is a lack of studies on the estimation of stature in the adolescent population. In this study, the statures are estimated only through the lengths of the fingers and phalanges, and thus cannot be directly compared with other studies. For Korean adults, the coefficient of determination of the stature estimation derived from multiple regression analysis was .425 for males and .418 for females. In this study, the values of the coefficient of determination derived from the multiple regression equation considering both fingers and phalanges were .659 for males and .529 for females, respectively. Especially, in the case of males, either the multiple regression equation derived through each finger or phalange had a higher coefficient of determination than .600. Therefore, in the Korean population, when estimating the stature through the length of the finger and phalange, the estimation of adolescent is more accurate than the adult. In addition, previous studies conducted on Koreans, it also considered measurement variables known to be highly correlated with stature such as hand length and palm length through various studies [3, 18]. However, in this study, only the length of the fingers and the phalange was used to design the estimation model of the stature. It can be very difficult to get the victim’s hand in undamaged form at the scene of a disaster or a murder. Therefore, it may be more effective to estimate the stature with only parts of a hand, such as the fingers and phalanges.

## Conclusion

The results of this study showed that there is a statistically significant relationship between the length of the fingers and the phalanges of adolescents and the stature. According to current studies, estimating stature from the length of the fingers and phalanges is reasonably accurate compared with studies of various ethnicities and Korean adults. It has been confirmed that different results are obtained from the results of estimating statures performed for adults because adolescent has a high potential for growth. Of course, in the case of compared Korean adolescents and adults in this study, it is the limit of this study that they do not target the same person. However, this is a massive study that needs to be done over a period of 5 to 10 years. Therefore, in order to generalize this study, it is necessary to carry out a future study to compare the results after re-measuring the subjects who were selected in this study after they became adults.

When adolescents become victims of crime or disasters, estimating their height or gender can be a major criterion for identification, since their biological profiling is less complete than in adults. Thus, once the age of the victim is determined, the biological profile of the victim can be established by applying a regression model derived from this study to estimate the stature. Various kinds of method for estimating statures through body parts must be constantly studied and tested, since identifying a cadaver that is severely damaged and part of a body is a challenge for many forensic specialists. This study suggests that the possibility of estimating the stature in the incompletely cadavered fingers and phalange is left. Through the results of this study, it is expected to be used as the basic data of the studies related to finger and phalange lengths of Korean adolescents.

## Data Availability

The datasets used and/or analyzed during the current study are available from the corresponding author on reasonable request.

## Abbreviations

1DL: Thumb finger length
1DT1L: Thumb finger distal phalange length
1DT2L: Thumb finger proximal phalange length
2DL: Index finger length
2DI1L: Index finger distal phalange length
2DI2L: Index finger middle phalange length
2DI3L: Index finger proximal phalange length
3DL: Middle finger length
3DM1L: Middle finger distal phalange length
3DM2L: Middle finger middle phalange length
3DM3L: Middle finger proximal phalange length
4DL: Ring finger length
4DR1L: Ring finger distal phalange length
4DR2L: Ring finger middle phalange length
4DR3L: Ring finger proximal phalange length
5DL: Little finger length
5DL1L: Little finger distal phalange length
5DL2L: Little finger middle phalange length
5DL3L: Little finger proximal phalange length
rTEM: Relative technical error of measurement
